# Supplementary material for: Are carbon emissions trading and green financial instruments synergistic? -Comprehensive quantitative research based on content analysis
Source: PLoS One. 2024 Mar 7;19(3):e0298601. doi: 10.1371/journal.pone.0298601 (PMC10919601; doi:10.1371/journal.pone.0298601)
Supplement: S1 Data — (ZIP) [file pone.0298601.s001.zip › Data/地级市Z_F工作报告.zip!/║■▒▒/╛ú╓▌╩╨/└┤╫╘╣1⁄2╓┌║┼í╛┬φ┐╦╩2╛▌═°í┐_2005.docx]

各位代表：

我代表市人民政府向大会作工作报告，请予审议，并请市政协各位委员和列席人员提出意见。

一、过去五年工作回顾

市二届人大一次会议以来的五年，是不平凡的五年。在省委、省政府和市委的正确领导下，在市人大、市政协的监督支持下，市政府坚持以邓小平理论和“三个代表”重要思想为指导，认真贯彻落实科学发展观，全面推进改革开放和现代化建设，基本完成预定的工作目标和任务。这五年，恰逢跨越世纪，千年更替，四大主要变化让全市人民倍受鼓舞：一是长江干堤全面加固，长期困扰荆州发展的洪水威胁得到根本治理，成为荆州发展史上的重要里程碑。二是“工业兴市”战略全面实施，荆州工业新区日渐成型，工业发展呈现新局面。三是城市建设全面加强，城市面貌大为改观，荆州跻身中国优秀旅游城市和省级园林城市行列。四是农村税费改革全面展开并取得历史性成就，农民人均纯收入突破3000元大关。

五年来，国民经济较快增长，综合实力显著提高。全市生产总值达到430亿元，年均增长8.6%，比“九五”时期高0.5个百分点；三次产业结构比由33∶38∶29调整为25∶43∶32，二产业比重每年上升一个百分点。工业经济整体素质明显提高，规模以上工业企业增加值年均增长11%。汽车零部件、纺织、家电、化工、食品加工等支柱产业竞争力明显提升，沙隆达、凯乐、洪城、博盈、天发、天颐等上市公司健康发展，楚源、拍马、恒隆、石油四机、神电、奥达、小天鹅、环宇等骨干企业发展势头良好，汽车电机、转向器和空调、农药、染料中间体、水工阀门、塑料管材、石油钻采机械等产品生产能力与技术水平居同行业领先地位。工业发展后劲明显增强，累计完成更新改造投资96.28亿元，年均增长14.5%；高新技术产业产值达到61.94亿元，增长近5倍。农业结构调整和农业产业化深入推进，农业基础地位进一步巩固，总产值达到193.59亿元，年均增长3.8%。粮食、棉花、油料等主要农产品产量继续保持全省领先地位，畜牧、水产、林业等特色优势产业得到长足发展。农产品优质品率提高，加工能力增强，农业产业化龙头企业总量规模居全省前列。第三产业快速发展，增加值达到137.87亿元，年均增长10.5%。商贸流通活跃，城乡市场进一步繁荣，成功举办全国油菜籽交易会，烟草、食品、药品、农资和成品油等市场整顿力度加大，大流通、大市场的商贸格局初步形成。全市社会消费品零售总额达到236.96亿元，年均增长10%，总量居全省第二位。文化旅游资源开发力度加大，房地产、信息服务业发展势头良好。财政金融运行平稳。财政收入达到23.66亿元，年均增长8%，公安县、松滋市财政收入突破3亿元；金融机构累计净投放140多亿元；积极化解金融风险，我市被评为全省最佳金融信用市。

五年来，改革开放取得重大进展，发展活力显著增强。企业改革全面展开。以产权制度改革、完善法人治理结构、健全国有资产监管体系为主要内容的改革攻坚战深入推进，国有工业企业改革和粮食、棉花、供销、商贸、物资等系统改革基本完成，全市企业改制面达90%以上，县域民营经济主体地位已经确立。农村改革稳步推进。实施农村税费改革，切实减轻农民负担，积极开展村级化债工作，农民生产积极性显著提高。财政体制改革初见成效。调整市县财政管理体制，推行收支两条线、政府采购、部门预算、国库集中支付改革，调动了各方面理财积极性。行政机构改革有序展开。顺利完成市县政府机构改革，大力推进行政审批制度改革，市级审批事项减少40%。启动了交通、文化、城建、卫生、教育等系统事业单位改革试点。全方位扩大对外开放。积极开拓国际市场，加快发展外向型经济，2004年外贸出口达到1.88亿美元，年均增长22.4%。坚持不懈地狠抓招商引资，累计利用外资2.39亿美元，年均增长13%。在荆投资的法雷奥、飞利浦、德克雷米、美洁时等大型跨国公司和小天鹅、华意、望春花、武商、中商等国内知名企业健康发展。海关、商检、口岸工作进一步加强，大通关优势初步显现。荆州港集装箱吞吐量年均增长41%，成为全省仅次于武汉的第二大港口。

五年来，投资和基本建设力度加大，城乡面貌发生显著变化。固定资产投资达到108.23亿元，年均增长8.9%。水利建设力度空前，累计完成投资68亿元，超过1999年前50年投资总和，实施长江干堤和荆南四河堤防整治加固，加强分蓄洪区、平垸行洪和移民建镇等工程建设，积极开展农田水利基本建设，防洪抗灾能力显著增强，千里江堤成为荆州一道亮丽的风景。交通建设超常规发展，累计完成投资50多亿元，荆州长江大桥建成通车，大桥连接线、襄荆高速公路、荆东高速公路、盐卡新港、东荆河大桥、宦子口船闸等重点工程建设进展顺利，建成乡村公路1600多公里，为荆州在江汉平原率先实现交通现代化奠定了坚实基础。电力保障能力不断增强，完成城乡电网改造投资10.18亿元，建成三峡交直流输变电荆州换流站，用电负荷居全省第二位。通信网络发展迅速，成为全省第二大信息网枢纽节点和传输中心。城市基础设施建设步伐加快，通过资本营运、争取开发银行贷款等途径，中心城区累计完成基础设施投资11.49亿元，重点建设荆沙大道、沙隆达广场、奥林匹克体育中心等基础设施，城市建成区范围扩大6.66平方公里。加强县市城区和7个国家级、9个省级重点镇建设，全市城镇化率达34.23%，平均每年提高2个百分点。

五年来，社会主义政治文明、精神文明进一步加强，社会事业显著进步。认真执行人民代表大会及其常委会的决议，主动接受人大及其常委会的法律监督和工作监督，接受人民政协和各民主党派、工商联、人民团体、无党派人士的民主监督。五年办理人大代表建议和政协委员提案1464件，办结率达到100%。群众性精神文明创建活动深入开展，荣获全国公益明星城和省级文明城市、双拥模范城等称号。社区服务功能不断增强，沙市区被列为全省社区建设示范区，洪垸社区被评为全国先进文化社区。文学艺术、社会科学、广播电视和新闻出版事业进一步发展，文物保护与利用得到加强。成功举办龙舟节、凤凰音乐节等活动，沙隆达广场被评为全国特色文化广场，“楚·虎座鸟架鼓”漆器图案入选中国和罗马尼亚联合发行的《漆器与陶器》特种邮票。加强人口与计划生育工作，我市荣获“全国计划生育系统先进集体”称号。建立土地招标、拍卖、挂牌出让制度，国土资源开发和保护规范有序。加强环境监测和污染治理工作，生态保护工作进一步加强。积极开展科技先进县(市)创建工作，累计争取和安排各级各类科技项目671项，登记科技成果220项。教育事业全面发展。实行以县为主的农村教育管理体制，“两基”成果得到巩固，教育质量稳步提高。支持长江大学的组建与发展，组建荆州职业技术学院和湖北中医药高等专科学校，高等教育规模居全省第二位。全民健身活动广泛开展，成功举办市二运会。认真贯彻实施《行政许可法》，建立健全行政执法责任制、违法责任追究制，规范执法行为，政府法制建设得到加强。大力推进政务公开，政府工作透明度进一步增强，政府网站受到欢迎。加强行政监察、审计监督和政务督查，切实纠正部门和行业不正之风，廉政建设和反腐败斗争不断深入。实施信访“句号工程”，真心实意为群众办实事、解难事，每年市政府向全市人民承诺办理的实事都较好完成。加强社会治安综合治理，深入开展“严打”整治斗争，依法惩治各种犯罪活动和“法轮功”等邪教组织，社会治安形势稳定。深入开展“四五”普法和法律援助服务。建立健全安全生产监管体系，有效防止重特大安全事故发生。国防动员、国防教育和民兵预备役工作扎实推进，二类预备役军官管理教育与分类动员工作走在全国前列。人民防空建设提前完成“十五”计划。民族、宗教、统计、编制、外事、侨务、地震、气象、档案、地方志等工作都有新的进步。

五年来，社会保障体系逐步健全，人民生活显著改善。社会保障覆盖面扩大，保障能力提高，全市参加基本养老保险人数达51.72万，五年净增13.52万人，累计发放养老金23.69亿元；职工医疗保险制度基本建立，参保人员达25.7万人；享受低保人口15.74万人，累计发放资金2.5亿元。就业再就业渠道进一步拓宽，累计帮助约18万名下岗职工实现再就业，其中4050人员6.45万人，城镇登记失业率控制在4.5%以内。扎实推进扶贫帮困和移民安置等工作，累计救助特困对象3.12万人。加强公共卫生体系建设，开展农村合作医疗试点，医疗服务水平进一步提高；建立突发公共卫生事件应急反应机制，取得防治非典、禽流感等重大疫病的胜利。加大血吸虫病综合防治工作力度，累计救治晚血病人6199人。城乡居民收入持续增长，城镇居民年人均可支配收入达到7600元，年均增长6.8%；农民年人均纯收入达到3002元，年均增长7%；城乡居民储蓄存款余额达到296.03亿元，年均增长26.9%。

2004年，全市上下认真贯彻国家宏观调控政策，沉着应对，趋利避害，继续推进“工业兴市”战略，加快农业结构调整，全面实现年初预期目标。主要特点是：

--实施“工业兴市”战略成效显现。积极发展符合国家产业政策的工业项目，千方百计化解煤、电、油、运和资金等要素瓶颈制约，大力支持有市场、有效益、有利于增加就业的企业加快发展，工业结构调整的主动性和企业自主增长能力增强，发展速度加快，主体地位更加突出。规模以上工业企业达到568家，比上年增加42家，增加值增长15.2%，增速为六年来最好水平；工业对GDP增长的贡献率达45.9%；工业企业入库税金达9.5亿元，占财政收入比重为39.9%，比上年提高1.5个百分点；完成更新改造投资25亿元，增长11.4%。工业经济的发展直接促进了就业状况好转，全市城镇单位从业人员自2003年实现七年来首次增长后，2004年新增1.27万人，中心城区领取失业保险金的人员由1.9万人降至0.9万人。招商引资工作体制和机制进一步完善，规模和水平明显提高，引资项目开工建设的达383个，实际到位资金34.19亿元。以荆州开发区为代表的省级省管开发区、工业园区继续保持快速发展势头，成为荆州工业发展的重要增长极。荆州工业新区累计完成基础设施投资3.9亿元，建成11.5公里东方大道，入园开工项目20个，其中4个项目建成投产。

--农业发展出现重大转机。充分利用国家扶农政策效应，积极发展粮食生产，加快农业经济结构调整，农业和农村经济发展呈现多年来少有的好形势。发放“两补”资金1.45亿元，降低农业税税率3个百分点，取消农业特产税，农民负担总额同比下降35%，人均负担下降45.3元。农民人均纯收入增加500元，七年来首次高于全省平均水平，增幅创九年来最好水平。全市优势产业带建设初见成效，区域特色开始显现。农业“项项增产，全面丰收”，粮食产量达到304.29万吨，增长17.4%，超额完成省下达的增产40万吨目标；棉花产量达到12.16万吨，增长19%；油料产量超历史，达到49.11万吨、增长29.3%；生猪出栏334.17万头，增11.01万头；水产63.47万吨，增2.93万吨；新增植树造林面积24.38万亩。监利、公安、洪湖、松滋四县市高产农田建设全面启动。天荣、天颐、吉象、银欣、金安、荆楚种业、万树、白云边等农业产业化龙头企业发展加快，农村中介组织健康发展，社会化服务能力增强，全市农村各类中介组织达到3500多家。

--改革开放迈出新步伐。整体推进农村税费改革、乡镇综合配套改革以及粮食流通体制、国有农场管理体制、农村卫生体制、血防体制、动物疫病防治体制等各项改革，第二轮土地延包工作顺利开展，监利、洪湖乡镇综合配套改革试点任务基本完成。企业改革扎实推进。市直企业产权制度改革基本完成，累计支付安置补偿费3.75亿元，转换职工身份达9万多名，占职工总数的95.6%。完成市政府机构改革任务。省管县财政管理体制得到落实。加大利用外资力度，实际利用外资6036万美元，增长9.7%；恒隆公司在美国纳斯达克上市，新西兰卡达维公司收购吉象公司，为在海外筹集发展资金打下良好基础。大力开拓国际市场，外贸出口增幅达56.9%，增长速度创历史最好水平。楚源、沙隆达、新生源、石油四机、德炎公司成为外贸出口领头雁，石首市成为全省外贸出口第一县市。与韩国江陵市缔结为友好城市，国际友好城市达到4个。

--城市建设力度加大。县市城区市政基础设施建设步伐加快。中心城区完成了工业新区、荆州古城、城南大学城等片区控制性详细规划编制，覆盖率达到80%。投资1.83亿元，改造江津路、北京路、南环路、荆州大道、沿江大道等主干道，荆沙大道续建工程步伐加快，启动城区水系改造工程，渍水问题正在抓紧治理。开展集中整治城区“脏、乱、差”行动，城市管理水平进一步提高。

--商业流通和房地产业蓬勃兴起。加强市场建设，成交额过亿元的市场发展到9个，蓝特商贸城和洪城商港年成交额超过20亿元。大力开拓城乡市场，农村消费额增长达7.6%，全市社会消费品零售总额增长速度创七年来最好水平。烟草市场整顿力度加大，销售税收大幅增长，达到9300万元。积极发展房地产业，规范房地产市场。中心城区实现政府土地收益1.68亿元；商品房开发规模和档次全面提升，销售面积增幅达20%以上。

--投资环境进一步改善。市政府行政服务中心正式组建运行，办事效率和服务水平进一步提高。继续实施荣誉市民制度，106名外来投资者被授予荣誉市民称号。贯彻实施《行政许可法》，清理行政许可事项，规范行政行为。建立市政府新闻发言人制度，促进政务公开。深入开展行风评议和机关作风建设，“三乱”现象得到遏制，治安环境改善，经济发展环境进一步优化。

--十件实事较好完成。一是新增城镇就业人员64625人，帮助双下岗职工家庭至少1人实现再就业8147人，分别超计划19625人和4147人。二是投资9000万元改造通乡公路300公里，投资2500万元改造通村公路500公里，完成预定目标。三是对农村特困人口31246人实施救助，投入4209万元建设改造福利院76所，农村“五保户”集中供养率达到84.7%，均超过计划目标。四是筹资1009万元资助贫困学生103256名，超计划43256名；筹资6428万元改造农村中小学D级危房123904平方米，超计划43904平方米。五是开展城区中小学周边环境综合整治，投资120万元对长江大学、荆州中学、沙市中学等重点部位实行电子监控。六是加强市县疾病控制中心、市紧急救援中心和传染病专科医院建设，完成投资3226万元。七是全市灭螺面积4.3万亩、治疗血吸虫病人6.8万人，救治晚血病人2466人，8个血防灭螺综合治理试点工程成效明显；公安、石首新型农村合作医疗试点参合农民分别达到43.8万人、28.6万人。八是集中整治城区大赛巷、春来市场等8个片区环境，拆除违章建筑15万余平方米；硬化城区小街小巷道路30条；开工建设城区豉湖渠和玉桥泵站，完成草市污水处理厂、北湖泵站土建工程。九是完成红金龙体育场改造，荆州体育场改造主体建筑完成90%以上。十是投资270万元改造新建江津路等主干道路灯，东方大道正全面安装太阳能路灯。

各位代表!过去的五年，是荆州综合实力不断增强、城乡面貌显著变化的五年，是改革取得重大进展、对外开放步伐明显加快的五年，也是人民生活不断改善、社会事业全面进步的五年。在出现诸多不利因素的情况下，特别是在防汛抗灾和抗击非典、禽流感的战斗中，我们经受了洗礼，积累了经验，锻炼了干部，凝聚了人心，增强了加快发展的信心和决心。这些成绩来之不易，是省委、省政府和市委正确领导的结果，是全市人民团结拼搏的结果，是社会各届人士大力支持的结果。在此，我代表市人民政府，向为荆州改革发展稳定做出积极贡献的广大干部群众，向全市人大代表、政协委员、各民主党派、工商联、人民团体和无党派爱国人士，向人民解放军、武警官兵和政法干警，向所有关心、支持荆州建设与发展的各界人士、海内外朋友，致以衷心的感谢和崇高的敬意!

回顾过去的五年，我们深切体会到，要做好政府工作，加快荆州发展，必须牢固坚持并始终把握好以下四点：

一是坚持紧扣第一要务，从荆州的实际出发确立发展思路。发展不够是荆州最大的实际。加快发展是解决荆州所有问题的关键。只有咬定发展不放松，大力实施“工业兴市”战略，全方位开展招商引资，锲而不舍地优化发展环境，才是顺应时代潮流、符合荆州实际、凝聚民心民智的正确发展道路。

二是坚持改革开放，运用市场经济的办法解决发展中的问题。 改革开放是加快发展的强大动力。只有以国有企业改革和农村税费改革为突破口，着力培育市场主体，加快发展民营经济，积极推进体制创新、机制创新、管理创新，充分发挥市场在资源配置中的基础性作用，才能不断增强经济发展的活力。

三是坚持科学发展观，实现经济社会协调发展。科学发展观是统领发展全局的重大战略思想。只有坚持以人为本，认真落实“五个统筹”，全面推进“三个文明”建设，加快构建和谐社会，才能真正走上可持续发展之路。

四是坚持依法行政，切实加强政风建设。依法行政是政府做好各项工作的根本保证。只有坚持依法行政，廉洁从政，执政为民，切实转变政府职能，转变工作方式和工作作风，求真务实，真抓实干，才能不断提高驾驭经济社会全局的能力和水平。

在看到成绩的同时，我们也清醒认识到，政府工作还有不少薄弱环节，经济社会生活中还存在一些突出的矛盾和困难。主要表现在：一是制约经济发展的诸多深层次问题尚未从根本上解决，体制改革、结构调整、转变经济增长方式等方面的任务仍很艰巨；二是农业基础较为脆弱，工业经济竞争力不强，县域经济、民营经济、外向型经济发展不够，农民增收、财政增收难度较大，经济运行的质量和效益有待提高；三是煤、电、油、运紧缺，部分企业尤其是中小企业资金不足，经济发展的瓶颈制约问题突出；四是就业再就业形势严峻，社会治安、城市管理和市场秩序不尽人意，影响社会稳定的诸多因素依然存在；五是政府自身建设尤其是行政能力建设、勤政廉政建设，以及改善发展环境，还需进一步加强，等等。造成这些困难和问题的原因，主要是我们思想解放不够，体制改革滞后，适应和驾驭市场经济的能力有限，以及结构调整难度很大。在今后的工作中，我们将高度重视，积极努力，认真加以解决。

二、今后五年发展构想

今后五年，是紧紧抓住重要战略机遇期、贯彻落实科学发展观的关键五年，也是实施“十一五”规划、加快荆州发展的关键五年。在新一轮经济结构调整和大发展中，荆州发展面临的总体环境比较有利。从外部环境看，国际国内经济持续增长，国际及沿海地区产业向内地呈现梯度转移趋势，国家“中部崛起”战略的实施和省委、省政府加快江汉平原与粮食主产区发展的决策，为荆州加快发展提供了重要机遇；从内部条件看，多年来特别是“十五”时期的发展为今后荆州发展打下了坚实基础，经济结构调整步伐加快，自主增长活力进一步增强，加之荆州独特的区位、丰富的资源、深厚的文化底蕴，荆州未来的发展具有充分的比较优势和后发优势。全市上下必须进一步增强加快发展的责任感和紧迫感，坚定信心，振奋精神，埋头苦干，奋发图强，推动荆州经济社会更快更好地发展。

今后五年政府工作的指导思想是：坚持以邓小平理论和“三个代表”重要思想为指导，全面落实科学发展观，深入推进“工业兴市”战略，加大体制改革、结构调整、招商引资和经济增长方式转变工作力度，加快新型工业化、农业产业化和城镇化步伐，不断增强城市综合竞争力，改善城乡人民生活，努力建设“和谐荆州”，促进全市经济社会全面、协调和可持续发展。力争主要经济指标增长速度高于“十五”时期水平，经济结构进一步优化，质量和效益明显提高，发展后劲显著增强，经济总量跃上新的台阶。

要实现上述目标，必须全面落实科学发展观，紧密联系荆州实际，把握好以下总体思路：

一是突出以新型工业化带动工业经济发展。要切实转变发展观念，调整发展思路，改变发展方式，大力发展高新技术产业，改造提升传统产业，以信息化带动工业化，积极发展循环经济，节约利用能源资源，走科技含量高、经济效益好、资源消耗低、环境污染少、充分发挥人力资源优势的新型工业化之路；二是突出以实施“工业兴市”战略带动经济发展。牢牢抓住工业经济发展这个“牛鼻子”不放松，扩大工业总量，调整工业结构，提高质量效益，以工业发展带动全市改革开放和一、二、三产业全面发展；三是突出以经济发展带动社会协调发展。经济发展是解决所有社会发展问题的前提和条件。要以经济建设为中心，一心一意谋发展，聚精会神搞建设，扎实做好“打基础、管长远”的工作，着力解决经济社会发展中的突出矛盾和问题，在经济发展的基础上推进社会事业全面进步；四是突出以城区经济发展带动农村经济发展。充分发挥中心城区和县市城区的龙头作用，增强城区产业集聚、要素集聚和人才集聚功能，提升综合竞争力，带动农村产业化和城镇化发展。通过全市上下的不懈奋斗，努力实现以下战略构想：

以农业为基础、以新型工业化为支撑，努力建设现代化工业城市--“新荆州”。农业是安天下的产业，水利是荆州天大的事，必须毫不动摇的巩固和加强农业的基础地位。依托荆州丰富的自然资源，坚持用工业理念谋划农业，加快推进农村经济结构的战略性调整，大力改善农村生产生活条件，着力培植优势产业和龙头企业，不断提高全市粮食综合生产能力、农产品市场竞争力、农业抵御自然灾害和市场风险能力，形成区域化布局、专业化生产、产业化经营、社会化服务的农村经济新格局，努力实现传统农业向现代农业的转变。工业是加快荆州发展的核心，必须坚定不移地强力推进“工业兴市”战略。紧紧围绕“工业兴市”三年见成效、五年翻一番的奋斗目标，狠抓项目兴工、园区兴工、改革兴工、科技兴工、环境兴工、实干兴工，围绕支柱产业抓招商，围绕产业集群抓招商，围绕工业园区抓招商，围绕骨干税源抓招商，努力形成以汽车零部件、纺织、家电、化工、食品加工为主体的优势产业群，以荆州开发区、荆州工业新区等省级省管开发区和工业园区为主体的产业集聚基地，以100家销售收入过亿元的重点企业为主体的具有较强支撑力和带动力的优势企业群体。

以文化旅游产业和现代教育为支撑，努力建设历史文化名城--“古荆州”。荆州是举世闻名的历史文化古城，具有浓厚的文化底蕴、独特的旅游资源和雄厚的科教基础。在严格保护、科学规划的前提下，坚持用产业化的思路、市场化的办法，整合文化旅游资源，建设以荆州历史文化景区为核心、以洪湖渔家生态景区、危水风景区、长江天鹅洲湿地景区为支撑的四大旅游景区，打好“三国牌”、“楚文化牌”、“名人牌”，努力把历史文化财富转化为现实生产力，把文化旅游产业培育成荆州经济的新亮点和支撑点，把荆州建设成为全国性、世界性的旅游目的地，争创中国最佳旅游城市。依托丰富的历史文化和教育资源，调整教育结构，深化教育改革，提高基础教育、职业教育和高等教育水平，提高市民人文素质和现代文明意识，提高城市文化品位，努力建设学习型城市，使荆州现代教育与历史文化相得益彰。

以优良的基础设施和生态环境建设为支撑，努力建设水乡园林城市--“美荆州”。围绕“滨江”、“水乡”、“园林”特色，高起点规划，高标准建设，高效能管理，加快推进城市功能建设和环境建设。坚持工业化与城市化相互促进，产业规划与城市规划相互协调，引导工业向园区集中、居住向社区集中。实施城市资本营运，建立多元化投资体制，加快构建和完善城市水、电、路、气等工程系统，工业区、商业区、办公区、居民小区等环境系统，文化体育、餐饮住宿、娱乐休闲、旅游景点等设施系统，单体设计、街景立面、建筑风格、城市色彩等标准系统，物业、交通、市场、治安、环卫等管理制度系统，不断完善城市功能，美化城市形象，彰显历史文化与现代文明相映衬的城市风格，展现“显城露水、古城秀水”的水乡园林城市风貌，打造“居住者自豪、投资者满意、外来人羡慕”的城市环境，积极争创国家园林城市。加快推进农村城镇化步伐，逐步形成以中心城区为龙头，以县市城区为骨干，以中心城镇为支点的城市群。

以经济实力和人才基础为支撑，努力建设江汉平原中心城市--“大荆州”。充分利用荆州的区位、交通、市场、人才和经济基础优势，积极参与长江三角洲及沿江经济带开放开发，主动融入泛珠江三角洲经济圈，使我市成为江汉平原中心城市。以构筑“大交通”、“大流通”为重点，进一步凸现荆州在江汉平原的集聚、辐射和带动作用。加快沪蓉国道、太澳高速荆州段和荆岳长江大桥、东荆河大桥等重点项目建设，加快江南、江北一级公路、县市城区连接高速公路网的高速通道建设，全面完善和提升公路交通网络；依托长江黄金水道，加快江汉航道建设，尽快将荆州盐卡港建设成为国家一类口岸和主枢纽港；积极争取“沪汉蓉”高速客运专线铁路武汉-荆州-宜昌段提前建设，争取荆岳铁路纳入国家铁路网络建设规划，加快完善荆州陆、水、铁、空相衔接的联运体系，在江汉平原率先实现交通现代化。改善和调整流通业态结构、组织结构和区域结构，加快商业街区、专业市场和流通龙头企业建设和发展，推进流通现代化进程，提升流通辐射力、带动力，努力把荆州建设成为江汉平原的商贸中心、物流中心和信息中心。充分发挥荆州人文荟萃的优势，在培养人才、吸纳人才、市场配置人才方面有新的突破，重点加强党政人才、企业经营管理人才、专业技术人才以及技能型人才队伍建设，为荆州经济社会发展提供智力支持。

三、2005年工作建议

根据省委、省政府和市委的总体工作部署，2005年全市经济社会发展的主要预期目标是：生产总值增长9%；全社会固定资产投资增长15%；社会消费品零售总额增长11%；外贸出口增长15%；财政收入增长8%；农民人均纯收入增长5%，城镇居民人均可支配收入增长5%；城镇登记失业率控制在4.4%以内；人口自然增长率控制在4.2‰以内。为实现上述目标，重点抓好以下八个方面的工作。

(一)以实施“工业兴市”战略为主导，增强工业整体竞争力。以提高工业经济运行质量和效益为中心，以培植企业核心竞争力为着力点，继续保持工业经济增长的强劲势头，确保全市规模以上工业增加值增长16%以上。

培育壮大骨干企业。因企制宜，分类指导，重点做大做强100家企业，即：年销售收入过10亿元的龙头型企业10家，其中楚源力争过30亿元，沙隆达、凯乐力争过20亿元；过5亿元的支撑型企业10家；过3亿元的潜力型企业20家；过亿元的成长型企业60家。建设一批重点项目，力争完成技改投入26亿元。对沙隆达公司高效低毒农药生产装置改造、恒隆公司50万套动力转向器生产线改造、环宇公司内燃机配件生产线改造等100个投资额过1000万元的技改项目，特别是法雷奥扩建、德克雷米300万台电机、凯乐短纤、沙市热电厂改造等16个重大项目，进一步落实项目责任制，加强跟踪协调和服务，争取尽早投产见效。对荆州火电厂、30万吨林浆纸一体化等重大后备项目，进一步加大向上争取的力度，争取尽早开工建设。加强项目策划、筛选、储备和开发，积极做好国债贴息等政策性项目的申报工作，力争有更多的项目挤进国家“笼子”。

培育壮大支柱产业。积极推进汽车零部件、纺织、化工、家电、食品加工等支柱产业的调整与发展，加快利用高新技术改造提升传统产业，逐步形成技术领先、竞争力强的产业群。集中精力办好荆州工业新区、荆州开发区和县市区工业园，进一步完善相关配套设施和服务，为要素集聚、产业集群搭建平台。荆州工业新区力争今年新增进园企业20家。支持石油四机、楚源、拍马、奥达等企业自办工业园，不断提高规模化、专业化水平。积极引导园区同类企业、产业链延伸企业簇群发展，形成有区域特色的板块经济。实施精品名牌战略，培育一批名牌产品，提升一批品牌知名度，改造一批传统名优产品，力争近年内培育国家级名牌产品2-3个，省级名牌产品8个。

培育壮大要素市场。针对土地、资金、能源等生产要素偏紧的状况，切实加强对工业经济运行的综合协调，确保项目建设和企业发展需要。加强政银企协调，充分发挥国有商业银行主渠道作用，积极支持其调整优化信贷结构，加大对重点项目和骨干企业的投入。支持市商业银行和农村信用合作联社等地方金融机构扩大规模，提高服务经济发展的能力。加强与开发银行的合作，积极引进其他国有股份制银行和外资银行，努力拓宽融资渠道。积极引导和支持企业上市，支持上市公司做好再融资工作，进一步做大做强。加强中小企业信用担保体系建设，完善“小额农贷”，推行“大额农贷”，为中小企业和农村经济发展提供资金保障。坚持集约用地，加快土地整理，盘活土地存量，增加单位投入强度，提高用地效益。加强电力调度，推广节能降耗等先进技术，保障重点企业生产和居民生活用电需要。

培育壮大企业家队伍。高度重视企业家的培养、引进和使用，尊重企业家，尊重纳税人，尊重创业者，激励各类企业经营管理人员在“工业兴市”中建功立业、多做贡献。

(二)以农民增收为核心，切实做好农业农村工作。

把解决“三农”问题作为各项工作的重中之重，加强农业综合生产能力建设，加快推进农业产业化经营，力争全市农业增加值增长4%。

认真落实党在农村的各项政策。认真落实农业税全免、粮食直补、良种补贴、农机具购置补贴和对产粮大县及财政困难县乡的转移支付等惠农扶农政策。继续抓好村级债务化解工作，强化涉农收费专项治理，规范“一事一议”制度，探索兴办村级公益事业的新途径。依法完善农村土地二轮延包工作，维护农民合法权益。

调整优化农业结构。在稳定粮食播种面积的基础上，着力提高单产水平，确保粮食产量稳定增长。高标准抓好16万亩高产农田建设，推进优质粮食产业工程。全面普及优质棉和优质油菜，提高种植效益。以生产小区和基地建设为重点，突破性发展畜牧、水产业，力争全市出栏生猪370万头，出笼家禽3800万只，水产品产量67万吨。组织农技人员进村入户，开展农业科技培训示范。扎实做好防汛抗灾工作，加强主要生产环节的组织和指导，确保增产增收。大力发展优质无公害农产品，全市力争建成农业标准化和无公害生产示范区12个。组织开展农民工技能培训，发展劳务经济，力争转移农村剩余劳动力新增5万人。

加快培育农村市场主体。以农产品精深加工为重点，加强农业招商引资和项目建设，壮大提升龙头企业规模和档次，力争年内新增市级龙头企业50家。鼓励龙头企业到农村建基地、签订单，与农户结成利益共同体。积极发展专业合作社、专业协会，扶持发展运销大户、种养大户，规范发展现代农业公司、私营农庄，提高农民组织化程度。

加强农村基础设施建设。多渠道增加投入，抓好荆南四河堤防加固、危水水库加固等重点水利工程建设，加强粮产区基本农田水利工程和四湖流域排灌体系建设，提高农业抗灾能力。加强农村道路、通讯、能源建设，建成通乡公路150公里，通村公路500公里，加快农村改水改厕和沼气推广等工作，改善农村生产生活条件。积极推广先进适用农业机械，力争全市农机化水平达到58%。推进产业扶贫，支持贫困乡村加快经济社会发展步伐。

(三)以发展商贸和文化旅游业为突破口，加快发展第三产业。

大力发展现代流通业。以世界500强首强企业沃尔玛公司进驻荆州为契机，加快推进商业结构调整和经营业态创新，提升荆州商业集聚、辐射功能。重点改造提升北京路商业中心区，建设中山路步行街、荆中路商业街，加快汽车交易市场、钢材市场、废旧金属与旧货交易市场建设，引导柳林洲水果副食批发市场和旧机动车交易市场进行资源整合，培育竞争力强的物流主体。发展代理制、连锁经营、电子商务等现代营销方式，提升传统商业档次。加强小城镇和农村生产生活资料连锁营销网络和配送系统建设，培育农村消费市场。同时，健全价格监测体系和预警机制，保持物价水平基本稳定。

加快发展文化旅游业。加强以三国文化和楚文化为主题的旅游开发，积极发展“红色旅游”，重点抓好荆州博物馆、东门景区改造，力争荆州博物馆和危水风景区分别成为5A、4A级景区；加强项目策划和招商引资，积极推进楚王陵森林公园、楚纪南城遗址公园等景区建设；启动市文物保护技术研究中心建设，为加强文物保护和发展文化旅游产业提供技术保障；加强旅游产品宣传促销，扩大荆州旅游品牌影响力。加快文化旅游资源整合，壮大广播电视、新闻出版、文化娱乐等产业，促进文化与旅游融合发展，提高文化旅游产业整体实力。

积极发展现代服务业。引导和规范房地产业健康发展，满足居民不同层次的住房需求。探索建立廉租住房制度，积极搞活住房二级市场，规范发展物业管理，加快中心城区危房改造步伐，不断改善市民居住条件。大力发展金融、保险、证券业，积极发展信息服务和会计、律师、咨询、经纪等中介服务业，鼓励和扶持社区服务业，进一步提高城市综合服务水平。

(四)以完善城市功能为主线，加强城市建设和管理。

加强城市规划工作。坚持以人为本和以城市“空间协调”为核心，做好城市总体规划修编工作，完善市域城镇体系规划、城市分区规划和专项规划，提高控制性详细规划覆盖率。加强规划管理，维护城市规划的严肃性，充分发挥城市规划在城市建设和经济社会发展中的引导和调控作用。积极做好历史文化遗存的保护工作。

加强基础设施建设。重点实施荆东高速、随岳高速监利段、荆州至汉南公路、洪湖东荆河大桥等工程，完成荆州长江大桥连接线、螺山干渠宦子口船闸一期、荆州物流中心站等工程建设任务，开工建设荆岳长江大桥，抓好荆州港规划完善和设施配套，继续做好荆岳铁路项目争取工作；实施城网改造续建、沙市热电厂燃气发电新建等工程，增强供电保障能力。加强中心城区功能建设和生态环境建设，抓好东方大道、荆沙大道、沿江大道等道路建设，进一步完善道路网络；继续建设豉湖渠抽升泵站和玉桥排水泵站，下大力解决渍水问题，改善城市水环境；建设草市污水处理厂，加强污水收集系统建设和雨污分流改造，开展护城河、西干渠疏浚整治，提高污水处理能力；进一步改造主干道两侧绿化设施，推动机关事业单位“开墙透绿”，提高城市绿化美化水平。加强县市城区交通、电力、供排水和污水处理设施建设及重点小城镇建设，提高城市化水平。

加强城市经营和管理。深化城建投融资体制改革，建立政府特许经营制度，通过公开招标、产权转让、使用权出让等途径，吸引各类投资主体参与市政公用设施的建设和经营，积极开展项目融资；加快市政公用行业市场化进程，加强户外广告管理，完成环卫、建安、园林绿化单位改制工作。积极开展城市管理综合执法试点，形成市区联动、部门齐抓共管的城市长效管理机制。进一步强化以社区为基础的城市管理工作，突出整治占道经营和“脏乱差”，改善城市人居环境。

(五)以建立市场经济体制为重点，扎实推进各项改革。

继续推进国有企业改革。以推进股权多元化为重点，继续深化国有及国有控股企业改革，上市公司和效益较好的国有及国有控股、参股企业全面完成国有职工身份转换，建立比较完善的法人治理结构和现代企业制度；加强改制企业的规范运作，妥善处理好资产处置和职工安置的问题；依法破产亏损严重、资不抵债的企业。加快建立健全国有资产授权监管、经营预算等相关制度，推进国有资产合理流动和布局调整，加强对重点企业尤其是国有产权交易的监管，确保国有资产保值增值。支持民营企业采取收购、兼并、参股、租赁等形式参与国有集体企业改组改制，消除制约民营经济发展的政策性、体制性障碍，支持民间资本进入法律法规未禁止的领域。实施民营企业成长工程，扶持100家骨干民营企业发展壮大。

继续推进财政管理体制改革。以建立公共财政为核心，积极推行部门预算，进一步扩大覆盖面；全面推进国库集中收付制度改革，市本级所有行政事业单位全面实行国库集中收付；继续深化政府采购制度改革，实行管办分离，进一步完善政府采购操作规范和运行机制；严格执行收支两条线管理规定，确保行政事业性收费、政府性基金、罚没收入等所有非税收入全部纳入财政预算管理。同时，大力开展增收节支活动，确保工资发放和重点项目支出。

继续推进乡镇综合配套改革。总结试点经验，全面稳步推进，年内基本完成全市乡镇综合配套改革任务。乡镇机关严格定员定岗，减少财政支出，减轻农民负担，提高执政能力，提高服务功能。加快事业单位人员分流，探索变“以钱养人”为“以钱养事”。继续深化农场改革。

继续推进事业单位改革。重点改革事业单位管理体制和用人、分配等制度，理顺政事、事企关系。力争年内基本完成城建、房管、交通、文化等系统事业单位改革，办好科技、教育、卫生、农业等系统事业单位改革试点。继续深化血防体制改革。积极探索运用市场的办法整合事业单位资源，增强事业单位发展活力。

(六)以招商引资为抓手，进一步扩大对外开放。

加大招商引资力度。加强招商引资责任制和目标考核，进一步完善招商引资的体制和机制。围绕延伸产业链和发挥比较优势，认真策划、论证、筛选一批项目，高标准建设项目库。坚持引进内资外资和引进管理、技术、人才并重，坚持“招商主体以企业为主，招商项目以工业为主，招商对象以沿海发达地区为主，内资外资以外资为主”，重点组织好赴江浙、香港和深圳“高交会”以及国外汽车零部件展等招商活动。加大招商项目的综合服务和协调督办力度，努力提高项目入园率、资金到位率和开工率。

切实优化发展环境。进一步深化行政审批制度改革，减少和降低收费项目和标准，营造低成本的投资环境。进一步加强行政服务中心建设，全面实行首问负责制、服务承诺制、审批联办制、集中收费制，严肃查处“三乱”行为，营造良好的服务环境。进一步支持金融机构做好不良贷款清收和处置工作，降低不良资产比重，开展最佳信用县市和最佳信用乡镇创建活动，营造诚实守信的信用环境。进一步整顿和规范市场秩序，加强食品、药品、烟草和生产资料等市场监管，营造公平竞争的市场环境。

努力扩大外贸出口。实施出口市场多元化战略，积极扩大机电、化工、纺织及农产品等优势产品出口，着力优化出口商品结构，鼓励企业自营出口，培植新的外贸增长点。加强口岸和“大通关”建设，提高外贸服务水平。

(七)以构建“和谐荆州”为目标，推动各项社会事业全面进步。

千方百计扩大就业和再就业。进一步加大就业再就业优惠政策落实力度，鼓励自主创业和灵活就业，强化劳动力市场建设与管理，将农村剩余劳动力转移就业纳入公共就业服务体系，大规模开展就业再就业培训，重点解决好“4050”人员再就业和“4555”人员社保援助问题，城镇登记失业率控制在4.4%以内。进一步扩大社保覆盖面，提高各类企业参保率，提高征缴率，力争全年扩面新增8.11万人，实现“两个确保”。以改制企业分流人员和断保人员为重点，抓好社会保险接续工作。继续抓好城市“低保”工作，妥善解决优抚对象以及特困群体的生活保障问题。

全面发展各项社会事业。加强以企业为主的科技创新体系建设，扶持一批高新技术企业做大做强；推动企业与科研机构、大专院校加强合作，加快科技成果利用和转化。优先发展教育事业，继续抓好“两基”工作，全面推进素质教育，巩固和提高农村义务教育水平。积极支持长江大学建设，支持湖北中医药高等专科学校和荆州职业技术学院建设新校区，加快发展职业技术教育和成人教育，鼓励民办教育发展。加强人才资源开发，落实人才优惠政策，形成培养人才、用好人才的新机制。加快疾病预防控制、突发性公共卫生事件医疗救治、重大疫情信息网络和卫生执法监督体系建设，办好公安、石首农村新型合作医疗试点，健全完善农村和社区卫生服务网络，全面提高医疗卫生服务质量和水平。深入开展爱国卫生运动。推进全民健身活动，办好市第三届运动会。

认真落实三项基本国策。扎实做好人口与计划生育工作，加强基层计划生育基础设施和服务体系建设，加大综合治理力度，稳定低生育水平，平衡新生儿性别比，提高出生人口素质。依法加强基本农田保护，加大经营性土地招标、拍卖、挂牌出让力度，规范土地市场，保护和合理利用国土资源。加大环境污染治理力度，严把项目环保评审关，发展循环经济，推进清洁生产。加强洪湖、天鹅洲湿地生态保护区建设。认真搞好全国第一次经济普查和人口抽样调查工作。精心编制“十一五”规划，力争荆州优势产业和项目进入国家、省“十一五”规划。

努力维护社会稳定。围绕创建“平安荆州”，以“命案必破”为重点，深入开展社会治安综合治理，完善社会治安防控体系，严厉打击各种刑事犯罪和黑恶势力，坚决铲除“黄赌毒”等丑恶现象。加强司法和国家安全工作。继续实施信访“句号”工程，规范信访秩序，积极探索建立解决信访问题的长效机制。扎实抓好安全生产和消防工作，防止重特大安全事故发生。

切实加强精神文明和政治文明建设。加强公民道德建设和科普宣传，全面完成“四五”普法任务，广泛开展群众性精神文明创建活动。认真执行市人大及其常委会的决议决定，自觉接受市人大的法律监督、工作监督，接受人民政协和各民主党派、工商联、无党派人士的民主监督，发挥新闻舆论监督作用，认真办理人大代表建议和政协委员提案，推进民主决策、科学决策。加强基层民主法制建设，完善村民、居民自治。依法开展国防教育、国防动员和民兵预备役工作，支持驻荆部队做好编制体制调整改革和军事斗争准备。积极开展争创“双拥模范城(县)”活动。加强军事设施保护和人民防空建设。贯彻落实党的民族宗教和侨务政策，依法保障妇女儿童、未成年人和残疾人的合法权益，重视和支持工会、共青团、社科联、文联工作，做好统计、编制、气象、档案、老龄、文史和无线电管理等工作。

(八)以提高行政能力为着力点，切实加强政府自身建设。

进一步加强学习。各级政府工作人员要认真学习“三个代表”重要思想和科学发展观，党员要积极参加“保持共产党员先进性教育活动”，树立科学的发展观、正确的政绩观，增强宗旨意识、公仆意识和服务意识；加强学习法律法规，提高依法行政意识；加强学习现代科技和行政管理知识，增强驾驭和指导经济社会发展的本领，努力建设学习型政府。

进一步加强行政能力建设。加快转变政府职能，推进政府管理创新，在改进经济调节和市场监管的同时，更加重视履行政府社会管理和公共服务职能，为市场主体创造良好的发展环境，为人民群众提供安居乐业的社会环境，努力建设服务型政府。认真贯彻执行《行政许可法》和国务院《全面推进依法行政的实施纲要》，严格实行执法责任制、考评制和执法过错追究制，加强行政复议、行政应诉、规范性文件备案审查和行政审批事项清理工作，努力建设法治政府。加快建立健全各种突发事件应急机制，提高保障公共安全和处置突发事件的能力。建立健全科学决策、民主决策和决策咨询制度，完善新闻发布机制，推进电子政务建设，提升政府网站功能，提高决策透明度和公众参与度。

进一步加强政风建设。大力弘扬求真务实精神，讲实话，重实干，办实事，求实效，坚决克服形式主义和官僚作风，坚决反对做表面文章、搞“形象工程”。继续下大力精简会议、文件，转变会风、文风，集中更多的时间和精力，解决实际问题，促进各项工作落实，提高政府工作效能。

进一步加强廉政建设。坚持从严治政，认真落实廉政建设责任制，加强领导干部任期经济责任审计，推进和完善行政服务中心、国库集中收付中心、综合招投标中心、经济发展环境投诉处理中心建设，深入推进纠风专项治理工作，切实从源头上预防和治理腐败。发扬艰苦奋斗的优良传统和作风，坚持艰苦创业、勤俭办一切事业。加强公务员管理，建设一支素质高、能力强、作风好的公务员队伍。

各位代表，努力为人民群众办实事，是各级政府应尽的职责。这里，我代表市人民政府郑重承诺，今年要继续为人民群众办好十件实事：(1)新增城镇就业岗位6万个，安置下岗失业人员4.3万人，统筹解决下岗职工、大中专毕业生和农村剩余劳动力就业问题。(2)继续搞好血防综合治理试点工程，及时治疗晚血病人，加强农村医疗卫生体系建设，筹资2500万元，对99家乡镇卫生院实施改造建设。(3)筹资680万元，建立城乡特困群众大病医疗救助体系，解决贫困群众大病救治问题；继续推进福星工程建设。(4)实施“放心菜”工程，全市建成3万亩无公害蔬菜生产基地，建立健全无公害蔬菜质量安全监测体系。其中中心城区筹资5000万元，建成无公害蔬菜生产基地1万亩，设立3个“放心菜”专供点。(5)筹资4000万元，实施“村村通”信息工程。(6)筹资5000万元，发展城乡有线电视用户10万户。(7)筹资1000万元，加强边远乡镇客运站场建设，实现乡乡通达客运班车。(8)完成五一路、梅台巷、民主街等3个片区、5万平方米危房改造任务，启动绿化村、喜雨街等2个片区、8万平方米危房改造工程；减免5000户最低收入者承租国有直管公房租金。(9)中心城区和县市城区学校接收12000名农民工子女入学接受义务教育。(10)筹资800万元改扩建市青少年宫，将其建设成青少年德育教育和开展文体、科普活动的重要阵地。

各位代表，荆州发展的前景光辉灿烂，富民强市的任务光荣艰巨。我们一定要紧密团结在以胡锦涛同志为总书记的党中央周围，紧紧依靠和团结全市人民，以更加饱满的热情、更加昂扬的斗志、更加务实的作风，同心同德，开拓创新，全面完成各项工作任务，为加快荆州经济发展、全面建设小康社会而努力奋斗！
